# Moral decision-making in real and sacrificial contexts: a pilot study on social cognition in mild cognitive impairment

**DOI:** 10.1186/s40359-026-04849-z

**Published:** 2026-06-30

**Authors:** Federica Alfeo, Paolo Taurisano, Antonietta Curci, Damiano Paolicelli, Tiziana Lanciano

**Affiliations:** 1 Department of Education, Psychology, Communication, University of Bari “Aldo Moro”, via Crisanzio 42, Bari, 70122 Italy; 2 Department of Translational Biomedicine and Neuroscience (DiBraiN), University of Bari ‘Aldo Moro’, Bari, 70121 Italy

**Keywords:** Decision-making, Morality, Social cognition, Mild cognitive impairment, MCI

## Abstract

Effective social decision-making requires understanding interpersonal dynamics involving moral content, a process supported by social cognition. When this ability is impaired, as in Mild Cognitive Impairment (MCI), individuals may struggle to act appropriately, reducing social engagement and accelerating decline.

The study investigated moral decision-making and social cognition in individuals with MCI and healthy controls (*N* = 40; 20 per group). Participants completed tasks assessing Theory of Mind (ToM), emotion attribution, social situation understanding, and moral/conventional distinctions. Moral decisions were measured using interactive scenarios (sacrificial, real-life moral, and neutral), recording choices and response times (RTs). Additional measures included cognitive reserve, social engagement, and perceived social isolation.

Results showed that individuals with MCI performed significantly worse than controls in identifying normative behaviours, with no group differences in other social cognition domains. Both groups made similar choices in moral decision-making tasks. However, individuals with MCI exhibited longer response times in morally salient scenarios, while no group differences emerged in neutral scenarios. This pattern may tentatively suggest greater processing demands in morally relevant contexts. Additionally, individuals with MCI reported lower levels of cognitive reserve and social engagement.

These findings underscore the importance of assessing moral decision-making in clinical populations to support autonomy and social inclusion in ageing.

## Introduction

### Social cognition and its influence on moral decision-making

The ability to make decisions in social contexts relies on the capacity to interpret complex interpersonal dynamics—a capacity supported by social cognition. This umbrella term encompasses a range of abilities essential for navigating the social environment and for creating, in humans, *“a shared world in which we can interact*” [1, p.727]. Research in this domain has shown that social cognition enables individuals to perceive and interpret interpersonal dynamics, construct knowledge informed by societal norms and values, and integrate both conscious and unconscious processes in understanding others [2].

Among the core components of social cognition are emotion perception and Theory of Mind (ToM). Emotion perception refers to the ability to recognize others’ emotional states through observable cues [3], whereas ToM involves the ability to attribute mental states—such as beliefs, desires, and intentions—to oneself and to others [4, 5]. Although conceptually distinct, these processes are closely related and may partially overlap, as the inference of others’ mental states often relies on the interpretation of emotional cues. Collectively, these abilities provide both cognitive and emotional understanding, offering a foundation for interpreting social and situational contexts—an essential prerequisite for producing effective judgments and making appropriate choices.

Social cognition not only allows individuals to understand others but also plays a crucial role in guiding moral decision-making. In everyday life, moral reasoning is often embedded within decision-making and involves evaluations related to justice, rights, and welfare—essentially, how people should treat one another. Moral judgments frequently emerge in situations that may involve harm, violations of autonomy, or the transgression of personal rights, and they typically concern actions that affect not only the decision-maker but also others. People generally distinguish between actions that are merely unwise and those that are morally unacceptable, and they are often highly motivated to act in ways that align with their moral beliefs—even though what is considered “right” or “wrong” often depends on the specific context [6].

This highlights the importance of situational evaluation, a process that is dependent on intact social cognitive abilities. Indeed, forming a moral judgment or making a choice requires, from the outset, the ability to accurately interpret the social and contextual circumstances in which a decision is made. When this capacity is compromised, individuals may struggle to recognize social cues, adopt others’ perspectives, or anticipate the consequences of their actions; this, in turn, may hinder their ability to identify the most appropriate course of action [7, 8].

Empirical evidence suggests that impairments in social cognition can lead to decisions that deviate from social or moral expectations, as individuals may fail to recognize the most contextually appropriate or socially acceptable responses [9, 10]. By integrating information about others’ mental states with an understanding of the consequences of actions and beliefs, components of social cognition help individuals evaluate what is right or wrong in a given situation [11], supporting the ability to navigate complex interpersonal situations and regulate social behaviour [12, 13].

Research has also begun to examine how moral evaluations change across the lifespan. In particular, older adults have been shown to adopt more severe moral judgments [14], to differ from younger adults in the distinction between moral and conventional violations [15] and to show a shift in moral reasoning from intention-based to outcome-based evaluations with advancing age [16]. These findings suggest that individual differences may influence social cognition and moral evaluation, underscoring the importance of systematically monitoring these variations and examining their potential impact on behaviour, including in populations that may be particularly vulnerable to cognitive or functional changes.

### Sacrificial scenarios and real-life conflicts

In many situations involving the moral domain, individuals are confronted with moral dilemmas—scenarios in which competing moral principles make it difficult to determine a single correct course of action [17]. In experimental research, hypothetical scenarios are widely used to explore moral decision-making and the factors that shape moral judgments and choices [18]. Among these, sacrificial moral dilemmas—in which causing harm to one individual may prevent greater harm to others—are particularly prevalent. These paradigms allow researchers to systematically manipulate contextual variables while eliciting moral conflict, as participants must navigate emotionally and cognitively demanding choices [19]. According to dual-process theory, responses in these dilemmas are thought to emerge from the interplay between controlled cognitive processes and automatic emotional reactions. Utilitarian judgments, which prioritize the greater good, have often been associated with more deliberative reasoning, whereas deontological judgments, which emphasize the inviolability of individual rights, are more frequently linked to intuitive emotional responses [20]. However, this distinction should not be interpreted as a strict one-to-one mapping. Rather, both types of judgments may involve a combination of cognitive and affective processes, with their relative contribution varying depending on the context and the specific features of the dilemma.

Despite their widespread use [21–25], the validity of these dilemmas has been debated. Some studies suggest that choices made in hypothetical sacrificial scenarios may not reflect real-life behaviour [26]. These concerns often stem from the assumption that such dilemmas can serve as indicators of moral personality or predictors of real-world action. However, their primary contribution lies in “*the contrast between cases […] Why such different answers? And what does this say about our moral thinking?”* [27, p.1].

It is true that moral decision-making in everyday life rarely involves life-or-death situations. Instead, individuals often face tensions between moral norms—which guide prosocial or principled behaviour—and personal desires, which may motivate more self-serving actions. These real-life decisions, though less extreme, can still evoke complex emotional, cognitive, and physiological responses [28]. While such realistic scenarios are less commonly used in the literature compared to classic dilemmas such as the “Trolley” or “Footbridge” dilemma [29, 30], they have the potential to engage individuals in situations that feel more familiar and personally relevant. Therefore, investigating both sacrificial and realistic dilemmas may offer a more comprehensive understanding of how people navigate morally significant situations in both abstract and everyday contexts.

### Cognitive and social challenges in mild cognitive impairment

Mild Cognitive Impairment (MCI) is a condition that lies between healthy cognitive aging and pathological decline. It is defined by objective impairment in one or more cognitive domains—including memory, attention, executive functioning, language, perceptual-motor skills, and social cognition—while functional independence is still largely preserved [31–33].

Individuals with MCI may follow different trajectories: some revert to normal cognitive functioning, others remain stable, while a proportion progresses to a neurocognitive disorder [34, 35]. Although prevalence estimates vary depending on demographic and clinical factors [36], it is approximate that 10–15% of the global population over age 65 is affected by this condition, underscoring the importance of early detection and ongoing clinical monitoring [37, 38].

Among the challenges associated with MCI, the generalized cognitive slowing [39] and decision-making difficulties represent a significant concern [for a review, see 40], as they can contribute to further cognitive and functional decline. In social settings, repeated difficulties in making appropriate decisions—especially those involving moral or interpersonal considerations—may lead to social exclusion, particularly when individuals are perceived as violating norms of fairness or reciprocity [41].

For those with MCI, the risk of social isolation is particularly acute. A growing body of research shows that individuals in early cognitive decline are often aware of their impairments and may fear judgment or stigmatization [42], which can lead to avoidant coping strategies such as social withdrawal [38]. Importantly, social engagement has been shown to protect against cognitive decline: meaningful relationships provide cognitive stimulation, whereas isolation increases vulnerability to deterioration [43–45]. More generally, variability in the progression of MCI appears to be influenced by lifelong factors (such as education, occupational activity, and leisure engagement), which contribute to cognitive reserve, defined as the capacity to cope with brain pathology through accumulated cognitive resources, and may help mitigate the impact of cognitive decline [46].

Taken together, these findings highlight the importance of identifying the mechanisms underlying decision-making difficulties in individuals with MCI, especially those involving social and moral reasoning, as well as the role of protective factors. Reduced social participation and limited social-cognitive stimulation may impair the processing of complex social situations, particularly when decisions are ambiguous or have significant social consequences. Because moral decisions require integrating emotional, interpersonal, and contextual information, limited social-cognitive resources may impact decision-making strategies. In contrast, greater cognitive reserve may support more adaptive processing and partially compensate for cognitive decline, improving decision-making efficiency in socially and morally relevant situations.

Although this issue is clinically and theoretically significant, no study to date has directly examined how social cognition impairments affect moral decision-making in individuals with MCI. This gap offers an opportunity for novel and impactful research.

## The present study

### Aim and hypothesis

Considering that moral decision-making relies on the ability to accurately interpret others’ intentions and integrate social and emotional cues, MCI represents an ideal model for exploring the relationship between social cognition and moral judgment. Individuals with MCI frequently show impairments across a range of cognitive domains—including, potentially, social cognition. However, the extent to which these difficulties affect moral decision-making remains unclear, as does whether different patterns emerge when individuals are confronted with sacrificial versus real-life dilemmas.

Based on these considerations, the present study aims to investigate social cognition—including ToM, emotion attribution, social situation analysis, and moral/conventional distinction—as well as moral decision-making in individuals diagnosed with MCI compared to healthy controls.

Using interactive, computer-based scenarios, the study assesses participants’ choices and response times (RTs) in both real-life and sacrificial moral dilemmas. Additionally, it explores the influence of individual differences in cognitive reserve, social engagement, and perceived social isolation on moral decision-making processes.

The study proposes the following hypotheses:

- H1: Individuals with MCI are expected to exhibit lower levels of social cognition compared to healthy controls.
- H2: Individuals with MCI are expected to show longer RTs during moral decision-making tasks—both compared to healthy controls and relative to neutral scenarios. In real-life dilemmas, they may be more inclined to prioritize self-interest, potentially reflecting reduced sensitivity to the social context. In sacrificial dilemmas, they are expected to show a greater tendency toward utilitarian responses, possibly due to a reliance on rule-based reasoning strategies when faced with situations potentially perceived as ambiguous.
- H3: It is hypothesized that individual differences in cognitive reserve, social engagement, and perceived social isolation will be associated with variations in RTs and moral choices, with potential differences between groups. These associations may reflect the role of cognitive reserve and social engagement in supporting cognitive flexibility and adaptive decision-making. Specifically, higher cognitive reserve is expected to be associated with faster RTs and a greater tendency toward morally driven choices (corresponding to deontological responses in sacrificial scenarios). In contrast, weaker social engagement and greater perceived isolation are expected to be linked to slower RTs and a greater inclination toward self-serving or utilitarian choices, possibly due to reduced cognitive and social resources available to cope with complex moral situations.

## Method

### Design

The study employed a between-subjects design, with Group (MCI patients vs. healthy controls) as the independent variable. The dependent measures included participants’ responses to moral dilemmas, comprising real-life scenarios (both moral and neutral, the latter serving as a control condition) and sacrificial scenarios, as well as measures of social cognition, cognitive reserve, social engagement, and perceived social isolation.

### Participants

The final sample included 40 participants: 20 individuals diagnosed with MCI (mean age = 70.8, SD = 9.65, range = 54–84; 10 females) and 20 healthy controls (mean age = 59.6, SD = 7.04, range = 54–81; 11 females). While the sample size was limited due to the practical challenges of recruiting clinical participants, the study was designed as a pilot investigation aimed at exploring preliminary trends and informing future research.

The experimental protocol lasted approximately 75–90 min and was administered across two sessions. Given the clinical characteristics of part of the sample, particular attention was paid to minimizing fatigue. Participants were offered short breaks when needed, and the experimenter monitored their level of engagement throughout the assessment.

All participants were native Italian speakers and took part on a voluntary basis. No financial or other incentives were provided. Informed consent was obtained from each participant prior to inclusion, and the study received ethical approval from the local Ethics Committee of the IRCCS Oncology Institute “Giovanni Paolo II” (Prot. 1772/CEL). MCI participants were selected based on comprehensive neurological evaluations, in accordance with diagnostic criteria established by Petersen and colleagues [47]. Exclusion criteria included a diagnosis of major neurocognitive disorder, the presence of psychiatric or neurological conditions, and the inability to provide informed consent. A control group of cognitively healthy individuals was recruited through a snowball sampling procedure for comparison (see Table 1).

**Table 1:** Sociodemographic characteristics of the control and MCI groups. The table reports categorical variables (gender and marital status) and continuous variables (age)

	**Category**	**Groups**	***n***	**% of Total sample**
Gender	Female	Controls	11	27.5 %
		MCI	10	25.0 %
	Male	Controls	9	22.5 %
		MCI	10	25.0 %
Marital Status	Single	Controls	1	2.5 %
		MCI	0	0.0 %
	Married	Controls	15	37.5 %
		MCI	13	32.5 %
	Divorced	Controls	2	5.0 %
		MCI	2	5.0 %
	Widowed	Controls	2	5.0 %
		MCI	5	12.5 %
			Mean	SD
Age	Controls		59.6	7.04
	MCI		70.8	9.65

### Procedure and measures

#### Screening

Participants diagnosed with MCI were recruited from the Policlinic Hospital, where they had undergone comprehensive neuropsychological evaluations conducted by specialist clinicians. Diagnoses followed Petersen and colleagues [47] criteria, based on objective evidence of cognitive impairment documented through standard clinical assessments. Prior to participation, all individuals underwent cognitive screening using the Montreal Cognitive Assessment (MoCA) to confirm that no undiagnosed cognitive impairments were present in the control group [48]. Only control participants with a MoCA score of 26 or higher were included. No participants were excluded after screening.

#### Social cognition assessment

Social cognition was assessed using the *Social Intelligence Battery* [49] (Table 2), which includes four tasks:

- ToM: Participants read short stories involving characters and were asked to adopt the protagonist’s perspective (13 items). A total ToM score (range: 0–13) was calculated, with higher scores indicating better ability to infer others’ mental states.
- Emotion attribution: Participants viewed a series of short scenes and were asked to identify the protagonist’s emotional state (58 items). Scores were calculated across seven emotional domains—sadness, fear, embarrassment, disgust, happiness, anger, and envy—with mean values ranging from 0 to 1. Higher scores reflect more accurate identification of target emotions.
- Social situations analysis: Participants evaluated whether the behavior described in each story was normal, a little strange, quite strange, or extremely strange (25 items). Three scores were obtained: identification of normative behaviors (mean score: 0–1); recognition of social violations (mean score: 0–1); and perceived severity of violations (range: 0–3).
- Moral/conventional distinction: Participants assessed behaviors typically observed in school settings, distinguishing between moral and conventional violations (12 items). For each category, three scores were calculated: whether the behavior should be allowed (range: 0–6); severity of the behavior (range: 0–60); and whether the behavior would be permitted in the absence of explicit rules (range: 0–12).

**Table 2:** Overview of cognitive, social, and moral decision-making measures. The table reports the type of outcome evaluated, the instruments used, the outcomes, and examples of items or dilemmas

Evaluation Type	Test	Outcome	Item Examples
Cognitive Reserve	Cognitive Reserve Index Questionnaire (CRIq)	- General cognitive reserve - Reserve based on education - Reserve based on working activity - Reserve based on leisure activities	—
Social Engagement	Lubben Social Network Scale	- General score of social engagement	—
Perceived Social Isolation	UCLA Loneliness Scale	- General score of loneliness and social isolation	—
Social Cognition	Social Intelligence Battery – ToM	- General ToM score	“*Late at night*,* Mrs. Bianchi is walking home. She doesn’t like walking home alone in the dark because she is always worried that someone might attack and rob her. Suddenly*,* a man appears from the shadows. He wants to ask Mrs. Bianchi what time it is*,* so he walks towards her. When Mrs. Bianchi sees the man approaching*,* she starts trembling and says*,* “Take my purse*,* but please don’t hurt me!”*” Q: Would the man be surprised by what Mrs. Bianchi said? (Yes / No) Why did she say that to him, given that he only wanted to ask her what time it was?
	Social Intelligence Battery – Emotion Attribution	- Seven scores across emotional domains: sadness, fear, embarrassment, disgust, happiness, anger, envy	“*Simone’s paintings were placed last in a competition.* *How will Simone feel in this situation*?” (Domain: sadness. Acceptable responses: sad, disappointed, sorrowful, distressed)
	Social Intelligence Battery – Social Situations	- Identification of normative behaviors - Identification of violations - Perception of violation severity	“*It was a hot summer day*,* and Giovanni enjoyed having his lunch in the nearby park. It had been quite warm in the office*,* so when Giovanni arrived at the park*,* he took off all his clothes and ate his sandwich naked*.” Q: How would you rate this behavior? ☐ Normal ☐ Somewhat strange ☐ Quite strange ☐ Extremely strange
	Social Intelligence Battery – Moral/Conventional Distinction	- Moral/conventional behaviors allowed - Severity of behaviors - Behaviors not allowed in absence of rule	“*A boy jumps on the school piano and starts breaking it with a hammer.” (Moral violation)* *“A student gets up and walks out of the classroom in the middle of the lesson.*” (Conventional violation) Q: Is it right for him to do this? (Yes / No) How severe is this on a scale from 0 to 10? Would it be right in a country with no law against it? (Yes / No) If the teacher allows it, would it be right? (Yes / No)
Sacrificial Scenarios	Footbridge-type moral dilemmas	- Composite score (utilitarian vs. deontological choice) - Mean RTs for moral decisions	e.g., “I am standing on a bridge above a railway track as a train approaches at high speed. On the track below, five workers are standing and will be hit if nothing is done. Next to me stands a large stranger; pushing him off the bridge would stop the train but result in his death.” Choices: (1) let five people die; (2) kill the single man.
Real-Life Scenarios	Realistic moral and neutral dilemmas	- Composite score for moral scenarios (moral vs. self-serving choices) - Mean RTs for moral and neutral scenarios	Moral: “*I want to board the subway train that is about to leave and that runs only every 15 min. Next to me on the platform is an old man with a bag of groceries. As I am getting on the train*,* he accidentally drops the bag on the floor*.” Choices: (1) Catch the train; (2) Help the man. Neutral: “*I am on a walk and passing by an ice-cream parlor. I decide to get two different kinds of ice-cream. The vendor asks me if I would like them in a cup or in a cone*.” Choices: (1) Cone; (2) Cup

#### Moral decision-making assessment: choice and response time

Moral decision-making was assessed using interactive video-based dilemmas in which participants faced classic sacrificial scenarios. In each scenario, a train was heading toward five people, and participants had to decide whether to let them die (a deontological choice, based on adherence to moral principles) or intervene by sacrificing one person to save the five (a utilitarian choice, based on maximizing overall well-being). Moral choices were coded as binary outcomes (0 = deontological; 1 = utilitarian).

The *sacrificial moral tasks* included two scenarios as developed in a previous study [25]: the classic Footbridge dilemma, in which participants were asked whether they would push a person off a bridge to stop the train, and a modified version, in which they could pull a lever to open a trapdoor, causing the person to fall onto the tracks. These dilemmas were used to collect both choice data and RTs. A composite response score (range: 0–2) was calculated by summing utilitarian responses across the two dilemmas, with higher scores indicating a stronger tendency toward utilitarian decision (responses to the two scenarios were collapsed into a single index due to the limited sample size and the lack of significant differences in exploratory analyses). Average RTs were also computed across the two scenarios to obtain a single RTs value for analysis.

The same response measures (choice and RTs) were recorded for a set of *real-life moral dilemmas* [28]. Participants were presented with 20 interactive video-based scenarios: 10 neutral and 10 moral. In the moral scenarios, they were asked to choose between a response guided by moral standards or one driven by personal desires. A composite moral choice score (range: 0–10) was calculated by summing responses aligned with moral standards, with higher scores reflecting a stronger tendency to act according to moral principles. Average RTs were also calculated for the 10 moral scenarios. RTs were also recorded for the neutral scenarios; however, no composite choice score was calculated, as these decisions did not correspond to theoretically meaningful categories (see Table 3).

**Table 3:** Descriptive and inferential statistics for study measures in the control and MCI groups. Reported values include means, standard deviations (SD), Mann–Whitney U statistics, p-values, and effect sizes (rank-biserial correlations). One-tailed tests were applied exclusively to directional, hypothesis-driven comparisons. Statistics were omitted for variables showing no variability. Measures included Theory of Mind (ToM), emotion attribution, social situation analysis, moral/conventional distinction, dilemma-related measures (choices and response times, RTs), cognitive reserve (CRIq), perceived social isolation (UCLA), and social engagement (Lubben Social Network Scale)

Domain / Task	Measures	M_Controls_ (SD)	M_MCI_ (SD)	U Statistic	*p*-value	Effect Size
ToM	ToM Score	10.40 (2.16)	9.90 (2.07)	162.0	0.151	− 0.190
Emotion Attribution	Sadness	0.52 (0.21)	0.62 (0.20)	134.5	0.965	0.328
	Fear	0.83 (0.13)	0.85 (0.14)	174.5	0.767	0.128
	Embarrassment	0.59 (0.22)	0.58 (0.27)	198.5	0.522	0.008
	Happiness	0.59 (0.22)	0.81 (0.20)	88.0	0.999	0.560
	Disgust	0.90 (0.19)	0.81 (0.31)	177.5	0.230	− 0.113
	Anger	0.39 (0.19)	0.47 (0.35)	181.5	0.698	0.093
	Envy	0.57 (0.38)	0.47 (0.38)	170.0	0.203	− 0.150
Social Situations	Normative behaviours.	0.94 (0.07)	0.85 (0.11)	99.0	0.002	− 0.505
	Recognition of violations	0.85 (0.11)	0.79 (0.20)	155.5	0.115	− 0.222
	Perceived severity	1.81 (0.39)	1.66 (0.45)	158.5	0.133	− 0.208
Moral/conventional distinction	Moral behaviours allowed	6.00 (00)	6.00 (00)	—	—	—
	Severity of moral violations	56.45 (3.20)	54.75 (8.38)	191	0.603	0.045
	Moral - Not allowed (no rules)	12.00 (00)	12.00 (00)	—	—	—
	Conventional behaviours allowed	5.90 (0.31)	5.70 (0.57)	169	0.106	− 0.155
	Severity of conventional violations	43.50 (7.39)	46.00 (8.21)	153	0.901	0.235
	Conventional - Not allowed (no rules)	10.55 (1.93)	10.30 (2.23)	197	0.465	− 0.018
Sacrificial dilemmas	Moral choice	1.30 (0.87)	1.50 (0.76)	176	0.234	0.120
	RT	908 (232)	1032 (107)	118	0.013	0.410
Real-Life dilemmas	Moral choice	5.20 (1.28)	5.45 (1.28)	174	0.779	0.132
	RT - Moral	396 (106.2)	459 (69.6)	133	0.036	0.335
	RT - Neutral	406 (112.5)	449 (61.0)	163	0.164	0.185
CRIq	Education	109.65 (9.26)	98.20 (15.04)	104.5	0.010	− 0.478
	Work	107.60 (19.56)	101.75 (28.29)	168.0	0.394	− 0.160
	Leisure	107.10 (17.01)	95.90 (24.22)	116.0	0.024	− 0.420
	Total Score	110.65 (13.10)	98.75 (23.55)	97.0	0.006	− 0.515
UCLA	UCLA Score	30.40 (4.83)	28.70 (6.14)	171.5	0.448	− 0.143
Lubben Social Network Scale	Total Score	16.05 (4.95)	11.70 (4.39)	104	0.010	− 0.480

#### Cognitive reserve, social engagement, perceived social isolation

Cognitive reserve was assessed using the Cognitive Reserve Index Questionnaire (*CRIq*), which provides a global index (range: 60–174) based on three domains: education, working activity, and leisure activities. Higher scores indicate greater accumulated cognitive reserve [50].

Social engagement was assessed using the *Lubben Social Network Scale* (range: 0–30), which assesses the size and quality of an individual’s social connections, with a particular focus on support from family and friends. Higher scores reflect stronger social support and greater engagement [51].

Perceived social isolation was measured using the *UCLA Loneliness Scale* (range: 20–80), a widely used instrument for assessing subjective feelings of loneliness. Higher scores indicate greater perceived isolation [52].

## Data analysis

Statistical analyses were conducted using Jamovi software (version 2.3; The Jamovi Project), which operates on the R statistical platform. Descriptive statistics were used to summarize participants’ sociodemographic and cognitive characteristics, as well as scores related to social cognition and moral decision-making. Continuous variables are reported as means and standard deviations, while categorical variables are presented as frequencies and percentages. Given the small sample size and the non-normal distribution of several variables, all inferential analyses were conducted using non-parametric tests.

Analyses were presented in the same order as the study hypotheses to allow readers to directly follow the logical progression from the hypotheses to the corresponding results:

To test H1, group comparisons were performed on components of social cognition—including ToM, emotion attribution, recognition of social situations, and moral/conventional distinction—using one-tailed Mann–Whitney U tests, in line with the directional nature of the hypothesis.

The same analytical strategy was applied to test H2, examining between-group differences in moral decision-making responses (both choices and RTs) across sacrificial and real-life moral dilemmas. In addition, an exploratory, non-directional within-group comparison was conducted in the MCI group to examine differences between RTs in moral and neutral real-life scenarios.

To test H3, two-tailed Mann–Whitney U tests were used to compare MCI and control participants on cognitive reserve (CRIq), social engagement (Lubben Scale), and perceived social isolation (UCLA). Additionally, Spearman’s rank-order correlations were computed to explore associations between these individual difference variables and moral decision-making outcomes (choices and RTs) separately within each group (MCI and controls). Given the large number of pairwise correlations tested, the false discovery rate was controlled using the Benjamini–Hochberg procedure applied to all p-values.

## Results

Preliminary analyses confirmed significant group differences in cognitive status. MCI participants demonstrated significantly lower MoCA scores compared to controls (U = 0.0, *p* < .001). A significant age difference between groups was also observed (U = 64.0, *p* < .001). Given the small sample size and the non-normal distribution of several variables, group comparisons were conducted using nonparametric tests (see Table 3).

### Social cognition assessment

In the Theory of Mind (ToM) task, no significant differences were observed between groups. A similar pattern emerged for the emotion attribution task, with no differences across the assessed emotional domains (sadness, fear, embarrassment, disgust, happiness, anger, and envy).

In the social situations task, significant group differences were observed only in the identification of normative behaviours (U = 99.0, *p* = .002, *r* = -.505), with participants with MCI performing worse than controls. No differences were found in the recognition of social violations or in the evaluation of their severity.

Analysis of the moral/conventional distinction task was limited due to low variability in participants’ responses. Both groups consistently rated moral violations as unacceptable (M = 6.00, SD = 0.00) and indicated that moral violations would not be permitted even in the absence of explicit rules (M = 12.00, SD = 0.00). No significant group differences emerged in the remaining measures, including severity ratings and conventional violation judgments (see Table 3).

### Moral decision-making assessment

In sacrificial dilemmas, no significant group differences were observed in the type of moral choices made (utilitarian vs. deontological), indicating that participants with MCI did not differ from controls in overall moral orientation. However, RTs differed significantly (U = 118, *p* = .013, *r* = .410), with MCI participants requiring more time to respond than controls.

In real-life moral scenarios, again, no significant differences were found in the moral choices made. However, participants with MCI exhibited significantly slower RTs than controls in the moral condition (U = 133, *p* = .036, *r* = .335), while no group differences emerged in the neutral condition (see Table 3).

Within the MCI group, RTs for moral scenarios were descriptively longer than those for neutral scenarios; however, this difference did not reach statistical significance (W = 132, *p* = .330).

### Link between cognitive reserve, social engagement, perceived social isolation and moral decision-making responses

Significant group differences were observed in the CRIq education subscale (U = 104.5, *p* = .010, *r* = -.478), the CRIq leisure subscale (U = 116.0, *p* = .024, *r* = -.420), and the total CRIq score (U = 97.0, *p* = .006, *r* = − .515), with control participants scoring higher in each domain. No group differences were found for the CRIq working activity subscale. Regarding social engagement, the control group reported significantly higher Lubben Social Network Scale total scores (U = 104.0, *p* = .010, *r* = -.480), while no significant differences were observed for perceived social isolation (UCLA scale) (see Table 3).

Spearman’s rank-order correlations were calculated separately for each group to assess associations between psychosocial factors (cognitive reserve, perceived social isolation, and social engagement) and moral decision-making outcomes. Prior to correction for multiple comparisons, some associations were observed, particularly involving the CRIq education subscale and both moral choices and RTs. After applying a false discovery rate (FDR) correction, no correlations remained statistically significant. Uncorrected results are reported for completeness (see Tables 4 and 5), as they may help inform hypotheses for future studies; however, they should be interpreted with caution.

**Table 4:** Spearman’s rho correlations (before applying a false discovery rate [FDR] correction) for the control group, examining the associations between moral decision-making outcomes (choices and response times [RTs] in sacrificial and real-life dilemmas) and individual difference variables, including cognitive reserve (CRIq total score and subdomains: education, work, leisure), perceived social isolation (UCLA), and social engagement (Lubben Social Network Scale). *p* < .05 is indicated by *

	Sacrificial dilemmas		Real-life dilemmas	
	Moral choice	RTs	Moral choice	RTs
CRIq – Education	-0.455*	0.358	0.515*	0.062
CRIq – Work	0.088	0.128	-0.085	0.008
CRIq – Leisure	-0.134	-0.228	0.113	-0.098
CRIq – Total score	-0.292	0.047	0.139	-0.004
Perceived social isolation (UCLA)	-0.058	0.097	0.037	-0.023
*Lubben Social Network Scale – Total score*	0.334	-0.316	-0.264	-0.215

**Table 5:** Spearman’s rho correlations (before applying a false discovery rate [FDR] correction) for the MCI group, examining the associations between moral decision-making outcomes (choices and response times [RTs] in sacrificial and real-life dilemmas) and individual difference variables, including cognitive reserve (CRIq total score and subdomains: education, work, leisure), perceived social isolation (UCLA), and social engagement (Lubben Social Network Scale). *p* < .05 is indicated by *

	Sacrificial dilemmas		Real-life dilemmas	
	Moral choice	RTs	Moral choice	RTs
CRIq – Education	-0.144	0.373	-0.281	0.488*
CRIq – Work	0.124	0.033	0.059	0.115
CRIq – Leisure	-0.071	0.205	0.200	0.073
CRIq – Total score	-0.086	0.264	0.036	0.196
Perceived social isolation (UCLA)	0.122	-0.021	-0.213	0.032
Lubben Social Network Scale – Total score	-0.135	0.247	-0.011	-0.101

## Discussion

The present study investigated social cognition and moral decision-making in individuals with MCI compared to healthy controls, offering insights into specific cognitive processes in a condition that lies between healthy ageing and cognitive pathology. We examined key aspects of social cognition—including ToM, emotion attribution, social situation analysis, and the moral/conventional distinction—skills essential for navigating interpersonal interactions and making morally informed decisions in everyday life. Additionally, the study explored moral choices and RTs in response to both sacrificial and real-life dilemmas, aiming to better understand the processes involved in moral decision-making. Correlational analyses were also conducted to examine whether cognitive reserve, social engagement, and perceived social isolation were associated with differences in decision-making responses.

In line with H1, which predicted lower social cognition abilities in MCI patients compared to healthy controls, results showed that individuals with MCI performed worse in the identification of normative behaviours. However, no significant differences emerged in the recognition of social transgressions or in the evaluation of their severity. This pattern suggests that individuals with MCI may struggle to identify behaviours considered typical or acceptable in social settings, perceiving them as unusual, while still being able to detect and judge violations of those norms. This finding is consistent with the notion that, because rules are essential for the effective functioning of social systems and serve to protect individuals from various threats, people tend to exhibit heightened sensitivity to norm violations, often detecting them almost automatically [53]. However, the human system of rules encompasses both universal, fixed norms and more flexible, context-dependent social conventions, which are shaped by situational factors [54, 55]. The recognition of normative behaviour—that is, behaviour considered appropriate for a given context—may require a nuanced interpretation of the situation and may be particularly demanding for individuals with MCI: although the MCI group performed comparably to controls in identifying and evaluating moral and conventional violations, they may have been more reluctant to classify certain behaviours as fully “normal”. This hesitation could stem from uncertainty about specific scenario details, which may not have been fully understood or integrated, leading them to interpret otherwise appropriate behaviours as potentially ambiguous or not entirely appropriate.

No significant group differences were observed in the other domains of social cognition. This finding is not unexpected, as deficits in social cognition among individuals with MCI may be subtle and variable, reflecting the heterogeneity of cognitive profiles in this population. Indeed, MCI is characterised by a wide range of cognitive impairments, with some individuals showing pronounced deficits in specific domains, while others maintain relatively preserved abilities [47, 56]. In particular, among the components of social cognition, ToM represents a debated area in the literature: when deficits are observed in individuals with MCI or Alzheimer’s disease, they tend to be relatively mild and are often secondary to broader cognitive impairments, such as reduced executive functioning or working memory capacity [12, 57, 58]. Emotion recognition is another domain marked by inconsistent findings: while some studies report impairments in individuals with MCI, others do not confirm such deficits, resulting in ambiguous and heterogeneous outcomes [59].

Our findings provide partial support for H2, which predicted that individuals with MCI would exhibit longer RTs during moral decision-making tasks, a greater tendency toward utilitarian responses in sacrificial dilemmas, and a preference for self-interest over moral norms in real-life scenarios. While no significant group differences emerged in the types of moral choices—neither in sacrificial nor in real-life dilemmas— MCI participants showed significantly longer RTs in sacrificial dilemmas and in real-life moral scenarios. Notably, in real-life scenarios, this slowing emerged in morally relevant decisions, while no group differences were found for neutral ones. This pattern partially aligns with previous findings on generalised cognitive slowing in MCI [39], but the fact that group differences emerged only in morally salient scenarios may also tentatively suggest that these contexts place additional processing demands on individuals with MCI. Nevertheless, this interpretation should be considered cautiously and warrants further investigation in larger samples.

Our findings also partially support H3. As expected, significant group differences emerged in cognitive reserve, specifically in the overall CRIq score and in the education and leisure subscales, as well as in social engagement, with the MCI group scoring lower than healthy controls. These results align with previous research suggesting that higher levels of cognitive reserve are typically associated with a reduced risk of developing MCI, which may explain why participants in the control group exhibited greater reserve in our sample [60].

Contrary to our expectations, no correlations between psychosocial factors (cognitive reserve, perceived social isolation, and social engagement) and moral decision-making outcomes remained statistically significant after correction for multiple comparisons.

Nevertheless, some preliminary patterns emerged at the uncorrected level that may warrant further investigation. In the control group, the education subscale of cognitive reserve was positively associated with morally guided choices in real-life scenarios and negatively associated with utilitarian choices in sacrificial dilemmas (indicating a lower tendency to endorse decisions that involve harming one individual to maximise overall outcomes). This pattern suggests that educational attainment may support norm-based reasoning in real-life contexts, in line with the role of education in socialisation and the internalisation of behavioural norms [61, 62]. At the same time, real-life dilemmas may capture not only morality in a strict sense but also broader altruistic or prosocial tendencies [63], as these constructs partially overlap. Within such contexts, individuals with higher education may be more attuned to socially shared expectations, promoting responses that are more norm-consistent and signal interpersonal sensitivity [64]. Differences between sacrificial and real-life dilemmas may reflect their structural and emotional divergence, with the two paradigms likely engaging partially distinct cognitive and affective processes [26]. In the MCI group, a potentially noteworthy finding again concerned the education subscale of cognitive reserve, which was associated with longer RTs in real-life scenarios. Individuals with higher educational reserve tended to take more time when making morally salient decisions, which may indicate a more deliberative and reflective approach to complex situations. As Fiedler and Glöckner [65] note, “*Judgments and decisions of any sort -including those that involve matters of morality- are not a matter of magic but result from processing of information*” (p. 139), However, in a population with cognitive decline, longer decision times raise an important question: when do they reflect deeper cognitive engagement, and when do they indicate emerging cognitive difficulties? One plausible interpretation is that increased RTs reflect compensatory mechanisms, where individuals rely on educational reserve to sustain performance despite cognitive impairment. In this way, cognitive reserve may serve as a protective buffer, allowing individuals to maintain decision-making by investing more time and effort in information processing. This strategy may help preserve decision-making abilities and potentially delay functional deterioration over time [66]. Although the associations between psychosocial factors and moral decision-making require cautious interpretation due to their exploratory nature, these findings, alongside results from other studies, offer valuable insights and highlight important questions for future research.

## Limitations

This study presents several limitations that should be acknowledged.

First, the sample size was relatively small and did not meet the threshold recommended by the a priori power analysis. This may have limited the statistical power of the analyses and reduced the generalizability of the findings. Additionally, the length and complexity of the assessment protocol—which included cognitive, social, and moral decision-making tasks—may have contributed to participant fatigue, potentially affecting attention, engagement, and response consistency over time. Furthermore, the two groups differed in mean age, which may have influenced some of the observed outcomes. We therefore recommend interpreting the results with caution. Future studies should use age-matched samples or statistically control for age.

Second, we did not include a comprehensive assessment of general cognitive functioning, nor did we differentiate among the various subtypes of Mild Cognitive Impairment as defined by Petersen and colleagues [47], such as amnestic vs. non-amnestic or single- vs. multiple-domain impairment. Given the heterogeneity of cognitive profiles within MCI, this lack of subgroup analysis may have masked important within-group differences.

Third, although the study included both real-life and sacrificial moral dilemmas, the two formats were not directly comparable. The real-life dilemmas involved choices between morally driven and self-serving choices (reflecting everyday decision-making contexts) and were greater in number. In contrast, the sacrificial dilemmas focused specifically on utilitarian versus deontological choices and were limited to two classic sacrificial scenarios (in line with common practice in the field).

These limitations indicate several directions for future research. Studies should use larger sample sizes, include comprehensive cognitive assessments, and consider the diversity of mild cognitive impairment subtypes to better reflect population heterogeneity. Researchers should also adopt standardized, ecologically valid decision-making assessments, such as real-life moral dilemmas that elicit both utilitarian and deontological responses. Reducing task complexity may help minimize participant fatigue and enhance data quality.

## Conclusive remarks

The present study investigated social cognition and moral decision-making in individuals with MCI compared to healthy controls, offering insights into cognitive processes associated with the transitional stage between healthy ageing and cognitive impairment. Key components of social cognition were evaluated, including theory of mind, emotion attribution, social situation analysis, and the distinction between moral and conventional norms. Moral choices and RTs were assessed in both sacrificial and real-life dilemmas. Additionally, the study investigated the potential influence of cognitive reserve, social engagement, and perceived social isolation on decision-making patterns.

The findings indicate that social cognition is largely preserved in MCI, except for a reduced ability to identify normative behaviours. This may reflect difficulty in consistently recognizing behaviours considered typical or acceptable, although individuals can still detect and evaluate norm violations.

In moral decision-making, individuals with MCI made similar choices to controls in both sacrificial and real-life dilemmas. However, compared to controls, they showed significantly longer response times in morally salient scenarios, while no group differences emerged in neutral conditions. This pattern may tentatively suggest greater processing demands during morally salient decisions, indicating that while moral decision-making outcomes appear preserved in MCI, they may require greater cognitive effort.

Regarding psychosocial factors, individuals with MCI showed lower cognitive reserve, particularly in the education and leisure domains, as well as lower levels of social engagement. While associations between these variables and moral decision-making did not remain significant after correction for multiple comparisons, preliminary patterns suggested that educational reserve may influence both moral choices and RTs.

To the best of our knowledge, this is the first comprehensive study to examine the relationship between social cognition, moral decision-making, and cognitive reserve in individuals with MCI compared to healthy controls. By analysing responses to both sacrificial and real-life dilemmas, the study enhances understanding of the cognitive mechanisms underlying moral reasoning in early cognitive decline and healthy ageing. These findings provide valuable insights and establish a foundation for future research to support moral functioning and autonomy in ageing populations, with implications for theory and targeted clinical interventions. 

## Data Availability

In line with principles of integrity and transparency, the study was preregistered on the Open Science Framework (OSF: https://osf.io/ra56b). All materials are available in the OSF repository (https://osf.io/3r7wg/files/osfstorage).
